# Modified inspiratory flow pattern - a tool for lung protective ventilation

**DOI:** 10.1186/2197-425X-3-S1-A309

**Published:** 2015-10-01

**Authors:** B Jonson, LW Sturesson, G Malmkvist, S Allvin, M Collryd, M Bodelsson

**Affiliations:** Lund University, Section of Clinical Physiology Department of Clinical Sciences Lund, Lund, Sweden; Lund University, Section of Anaesthesia and Intensive Care, Department of Clinical Sciences Lund, Lund, Sweden

## Introduction

Lung protective ventilation, LPV, is recommended for ever wider patient populations, even those without primary lung disease. Low tidal volume ventilation is a first-hand remedy for LPV that is facilitated by dead space reduction. Inspiratory flow pattern affects gas exchange. In ARDS patients, it was recently shown that a long period during which inspired tidal gas is in contact with resident alveolar gas, mean distribution time, MDT, as well as a high end inspiratory flow, EIF, immediately before interruption of inspiration augment exchange of C02 by reduction of airway and alveolar dead space.

## Objectives

The hypothesis was tested that MDT and EIF affect C02 exchange in man without lung disease in principle as in ARDS. The study should provide guide lines in support of LPV at health as well as in lung disease.

## Methods

During preparation for cerebral surgery in 8 patients without lung disease, breaths with 20 inspiratory wave forms were delivered with different inspiration time, T_I_, post inspiratory pause time, T_P_, and shape in form of ramps with increasing or decreasing flow rate. Signals for flow rate and C02 were analysed in the format of the single breath test C02 from which the volume of C02 eliminated during each ordinary or modified tidal breath, V_T_C02, was derived. Changes of V_T_C02 were explained by changes in airway dead space and by effects on intra-alveolar gas mixing inversely reflecting alveolar dead space.

## Results

In relation to ordinary breaths with T_I_ 33%, T_P_ 10% and square inspiratory flow, the change of C02 volume exchanged per breath, DV_t_C02%, varied with MDT and EIF: DV_t_C02% = 17 · lnMDT + 13 · EIF +1.4

In each subject the influence of lnMDT and EIF was highly significant. Positive effects on C02 exchange by longer MDT and higher EIF reflected both a reduced airway dead space and improved intra-alveolar gas mixing indicating a reduced alveolar dead space.

Modification of inspiratory flow pattern could increase V_T_C02 by 17% at constant inspiratory flow and 27% at increasing inspiratory flow. The most efficient way to modify inspiration was to prolong T_P_ at the expense of a shorter T_I_ and eventually a shorter expiratory time. The more efficient C02 exchange allows a reduction of tidal volume by about 10%.

## Conclusions

Modification of inspiratory flow pattern, particularly by a longer T_P_, is a tool for more efficient C02 exchange that can easily be applied for lung protective ventilation at reduced tidal volume in patients without or with lung disease.
